# Supplementary figures and images for: In Vitro Release of Glycyrrhiza Glabra Extract by a Gel-Based Microneedle Patch for Psoriasis Treatment
Source: Gels. 2024 Jan 23;10(2):87. doi: 10.3390/gels10020087 (PMC10887857; doi:10.3390/gels10020087)

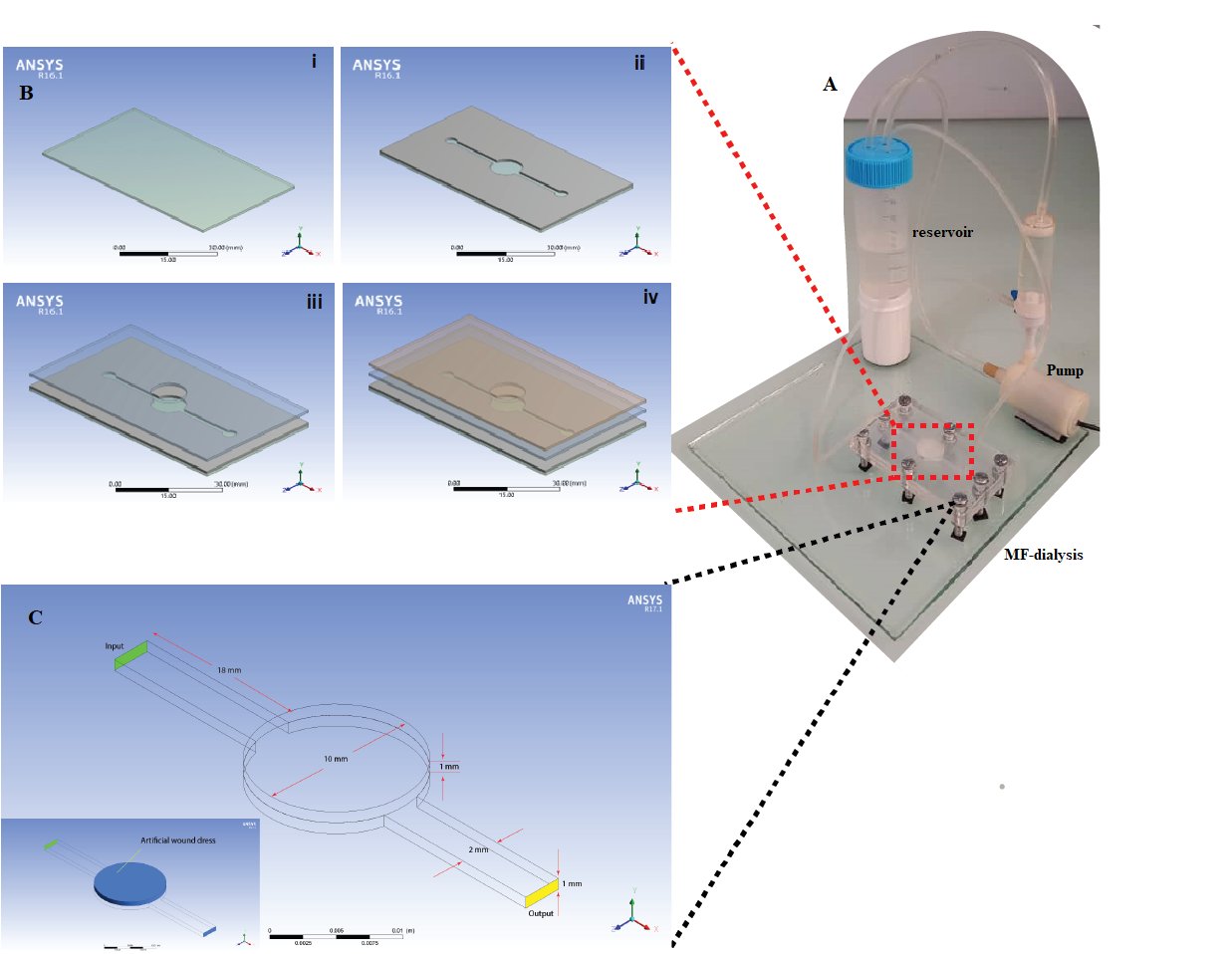

Supplement: Supplementary file 1 [file gels-10-00087-s001.zip › gels-2509159-supplementary.png]
